# Invasive Macrophytes Control the Spatial and Temporal Patterns of Temperature and Dissolved Oxygen in a Shallow Lake: A Proposed Feedback Mechanism of Macrophyte Loss

**DOI:** 10.3389/fpls.2017.02097

**Published:** 2017-12-08

**Authors:** Maria P. Vilas, Clelia L. Marti, Matthew P. Adams, Carolyn E. Oldham, Matthew R. Hipsey

**Affiliations:** ^1^UWA School of Agriculture and Environment, University of Western Australia, Crawley, WA, Australia; ^2^Sustainable Engineering Group, Faculty of Science and Engineering, Curtin University, Bentley, WA, Australia; ^3^School of Chemical Engineering, University of Queensland, St Lucia, QLD, Australia; ^4^School of Civil, Environmental and Mining Engineering, University of Western Australia, Crawley, WA, Australia

## Abstract

Submerged macrophytes can have a profound effect on shallow lake ecosystems through their ability to modify the thermal structure and dissolved oxygen levels within the lake. Invasive macrophytes, in particular, can grow rapidly and induce thermal gradients in lakes that may substantially change the ecosystem structure and challenge the survival of aquatic organisms. We performed fine-scale measurements and 3D numerical modeling at high spatiotemporal resolution to assess the effect of the seasonal growth of *Potamogeton crispus* L. on the spatial and temporal dynamics of temperature and dissolved oxygen in a shallow urban lake (Lake Monger, Perth, WA, Australia). Daytime stratification developed during the growing season and was clearly observed throughout the macrophyte bed. At all times measured, stratification was stronger at the center of the macrophyte bed compared to the bed edges. By fitting a logistic growth curve to changes in plant height over time (*r*^2^ = 0.98), and comparing this curve to temperature data at the center of the macrophyte bed, we found that stratification began once the macrophytes occupied at least 50% of the water depth. This conclusion was strongly supported by a 3D hydrodynamic model fitted to weekly temperature profiles measured at four time periods throughout the growing season (*r*^2^ > 0.78 at all times). As the macrophyte height increased and stratification developed, dissolved oxygen concentration profiles changed from vertically homogeneous oxic conditions during both the day and night to expression of night-time anoxic conditions close to the sediments. Spatially interpolated maps of dissolved oxygen and 3D numerical modeling results indicated that the plants also reduced horizontal exchange with surrounding unvegetated areas, preventing flushing of low dissolved oxygen water out of the center of the bed. Simultaneously, aerial imagery showed central dieback occurring toward the end of the growing season. Thus, we hypothesized that stratification-induced anoxia can lead to accelerated *P. crispus* dieback in this region, causing formation of a ring-shaped pattern in spatial macrophyte distribution. Overall, our study demonstrates that submerged macrophytes can alter the thermal characteristics and oxygen levels within shallow lakes and thus create challenging conditions for maximizing their spatial coverage.

## Introduction

Submerged macrophytes are often considered key components of shallow lake ecosystems due to their positive impact on water clarity and water column nutrient loads (Phillips et al., 2016). Introduced invasive macrophyte species, however, may represent a threat to shallow lake ecosystems due to their excessive growth and fast colonization rates (Hussner et al., 2017). By establishing dense canopies, invasive macrophytes can substantially alter water column mixing, allowing for thermal stratification to develop during the daytime (Herb and Stefan, 2004). Thermal stratification can in turn strongly influence the chemical and biological characteristics of shallow lakes (Branco et al., 2005; Andersen et al., 2017a). However, the effect of submerged macrophytes on the thermal characteristics of shallow lakes remains largely unexplored.

Submerged macrophytes can promote thermal stratification by reducing turbulent kinetic energy (TKE) and attenuating solar radiation (Herb and Stefan, 2004; Andersen et al., 2017b). Their impact on thermal stratification depends on canopy height relative to water depth, biomass and density as these attributes are inversely related to the mixed layer depth (Herb and Stefan, 2004; Coates and Folkard, 2009). By inducing thermal stratification, submerged macrophytes impede vertical transport of gasses, dissolved and particulate materials and influence the oxygen levels at the sediment-water interface (Caraco and Cole, 2002). Hypoxic conditions have been reported during daytime stratification in shallow turbid lakes (Branco and Torgersen, 2009), particularly if the lakes are fetch-limited and have reduced wind exposure. By facilitating oxygen depletion at the sediment-water interface, submerged macrophytes can induce nutrient release from the sediments (Boros et al., 2011; Vilas et al., 2017b), which is often enhanced by increased deposition of organic matter (Barko et al., 1991), and by the shallow depth of the systems they inhabit. These released nutrients may promote phytoplankton production, which could supply sediment organic matter that would further enhance bottom anoxia. Furthermore, oxygen depletion can negatively affect the macrophytes by impeding metabolic performance (Sand-Jensen et al., 2015) and facilitating organic matter degradation pathways that accumulate potentially phytotoxic compounds such as sulfide and iron (II) (Lamers et al., 2013). The onset of thermal stratification could therefore have a large impact on water quality, sediment properties and ultimately on vegetation dynamics.

*Potamogeton crispus* L. is an invasive submerged macrophyte that has established monotypic beds in lakes and rivers throughout the world (Bolduan et al., 1994). It has been reported to grow throughout the year in rivers and cold ponds (Kunii, 1989; Riis et al., 2003). However, *P. crispus* is generally considered to be a winter annual with environmental cues influencing its phenology (Chambers, 1982; Valley and Heiskary, 2012). Strong thermal gradients have been recorded in *P. crispus* beds (Leoni et al., 2016; Vilas et al., 2017b). By inducing thermal stratification *P. crispus* can promote conditions suitable for nitrogen-fixing cyanobacteria (Vilas et al., 2017b). Therefore, understanding the effects of *P. crispus* on the thermal characteristics of shallow lakes is of relevance for selecting management strategies of this species.

The aim of this study is therefore to assess the effect of the submerged macrophyte *P. crispus* on the spatial and temporal dynamics of temperature and dissolved oxygen in a shallow urban lake. Specifically, we integrate high-resolution field data and numerical modeling to address the following questions: how do seasonal changes in macrophyte attributes impact thermal stratification? What is the minimum macrophyte height needed to trigger thermal stratification and how do other macrophyte attributes affect this threshold? How does thermal stratification influence the diurnal oxygen dynamics? In line with these results, we propose a conceptual model outlining a potential plant-stratification-dieback feedback that has not been previously described.

## Materials and Methods

### Study Site

**FIGURE 1:**
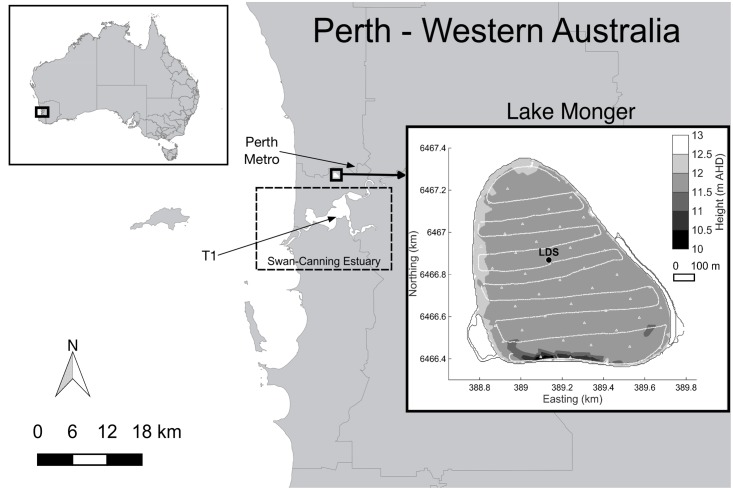
Lake Monger location and bathymetry in meters Australian Height Datum (m AHD). The location of the moored station (LDS), the meteorological station (T1), Perth Metro station and typical towing F-probe transects (continuous lines) and profiling stations (triangles) are also shown. Rainfall, relative humidity, short wave radiation, and net total radiation were sourced from station T1 and air temperature data from Perth Metro station.

Lake Monger is a eutrophic shallow lake located in urban Perth, Western Australia (**Figure 1**). It has a total surface area of 68.2 ha and its bottom boundary is on average at 11.89 m Australian height datum (AHD). The lake mean water depth varies seasonally from 1.2 m in spring to 0.3 m in autumn and the lake overflows to the Swan-Canning River Estuary when mean water depth exceeds 1.1 m. Lake Monger is colonized by dense stands of the submerged macrophyte *P. crispus* which forms monotypic beds throughout spring and summer (Vilas et al., 2017a). *P. crispus* was first documented in the lake in 1988 (Lund, 1992) and has been increasing in spatial extent and density during the past 6 years (R. Bowman, 2016, pers. comm., 22 July). In Lake Monger, *P. crispus* typically initiates growth in the middle of the lake by the end of winter, which is much later than reported elsewhere (Rogers and Breen, 1980). *P. crispus* then spreads toward the lake edges throughout spring and summer, and dies back toward the end of summer, starting in the center, after the top of the canopy reaches the water surface. Ring-shaped pattern formation has therefore been observed in this lake (Vilas et al., 2017a). *P. crispus* has a large influence on the water quality of the lake. The interested reader is referred to Vilas et al. (2017b) for details on Lake Monger’s water quality.

### Field Measurements

Field data were collected between November 2014 and January 2015, and between November 2015 and January 2016, covering two *P. crispus* growing seasons.

The lake thermal structure was continuously monitored by a Lake Diagnostic Systems (LDS, Imberger, 2004) station deployed near the center of the macrophyte bed (**Figure 1**). The LDS station was equipped with a fast response, high precision thermistor chain, measuring water temperature at 0.2, 0.4, 0.6, 0.8, and 1 m below the water surface, and wind speed and direction sensors located at 2 m above the water surface. The LDS station collected data every 30 s, in both growing seasons. Rainfall, relative humidity, short wave radiation, and net total radiation were also recorded at 2 m above the water surface by a LDS located at station T1 in the Swan-Canning River Estuary ∼7 km south of the lake (**Figure 1**). Data from this station were available for the first growing season and were collected every 30 s. Air temperature data were sourced from the Australian Bureau of Meteorology (Perth Metro station) and were available at 15-min intervals.^1^ Net heat fluxes were calculated using short wave radiation, total net radiation, wind speed and relative humidity recorded at station T1, air temperature recorded at Perth Metro, and surface water temperature recorded at the LDS station, as described by Verburg and Antenucci (2010).

In the first *P. crispus* growing season (November 2014–January 2015) a multi-parameter probe (Hydrolab DS 5X) was deployed at the LDS station for 2 week-long periods (8–15 November 2014 and 23–30 December 2014) to measure dissolved oxygen concentrations (DO, optical sensor, accuracy: 0.2 mg/L). In addition, a multi-parameter probe (Hydrolab Mini Sonde 4a) was deployed at the LDS station to measure redox potential (accuracy: 20 mV) and DO (optical sensor, accuracy: 0.2 mg/L) between 22 and 27 January 2015. Both multi-parameter probes were positioned at approximately 0.1 m above the lake sediments and sampled every 20 min. A fine-scale profiler (F-probe, Imberger and Head, 1994) was also used on 28 January 2015 to measure any spatial variability in DO (accuracy: 0.2 mg/L) and water temperature (accuracy: 0.001°C) across the lake. The profiler was deployed in free-falling mode (from the surface down to the lake bed) at selected stations placed approximately 100 m apart (**Figure 1**). The F-probe collected data at a rate of 50 Hz with a drop velocity of approximately 0.1 m s^-1^, yielding a vertical resolution of 2 mm.

In the second *P. crispus* growing season (November 2015–January 2016), two diurnal field experiments were carried out to assess the spatial and temporal patterns in temperature, DO, and turbidity over the diurnal cycle. The first diurnal field experiment took place on 4 November 2015 when above-ground plant biomass was low and canopies were short. The second diurnal field experiment took place on 7 January 2016 when above-ground biomass was large and canopies were tall. During these experiments the F-probe was used to measured DO, water temperature and turbidity (accuracy: 0.1 NTU) and was deployed from a boat in both towing and free-falling mode depending on the height of submerged macrophytes. When the macrophytes did not reach the lake surface, the profiler was towed approximately 0.25 m below the water surface over a series of west-east transects spaced ∼100 m apart (**Figure 1**). Transects and profiling stations were chosen to cover spatial gradients and GPS coordinates were recorded.

In both growing seasons, total above-ground macrophyte biomass at the LDS station was estimated by sampling a quadrant of 0.1–0.2 m^2^ (depending on the plant density) at biweekly or triweekly intervals. Plants within the quadrant were uprooted with a rake. Duplicate or triplicate biomass samples were collected and transported to the laboratory where they were washed and dried at 60°C to a constant mass and then weighed. Canopy height was measured at three randomly chosen points around the LDS station. Submerged aquatic vegetation mapping was done through visual analysis of aerial photography obtained from Nearmap^®^^2^ in conjunction with geo-referenced movies recorded with a 10 megapixel underwater digital camera along the west-east transects (**Figure 1**).

### Numerical Modeling

The 3D Estuary, Lake and Coastal Ocean Model (ELCOM, Hodges et al., 2000) was used to assess the effect of submerged macrophytes on thermal stratification and water exchange. ELCOM simulates 3D variations in velocity, temperature, salinity and concentration of conservative tracers in space and time by numerically solving the hydrostatic, Boussinesq, Reynolds-averaged, Navier Stokes and scalar transport equations (Hodges et al., 2000). The free-surface evolution is modeled by vertical integration of the conservation of mass equation applied to the kinematic boundary condition. The free-surface height in each column of grid cells moves vertically through grid layers as required by the free-surface evolution equation. The heat exchange through the water surface is governed by non-penetrative (longwave radiation, sensible heat transfer, and evaporative heat loss) and penetrative (shortwave radiation) components. Non-penetrative components are introduced as sources of temperature in the surface mixed layer, whereas penetrative effects are introduced as source terms in one or more grid layers according to exponential decay and an extinction coefficient (Lambert-Beer law).

The model simulates the effect of submerged macrophytes on the hydrodynamics by applying a macrophyte drag coefficient *C*_D_ (no units) and a macrophyte light extinction coefficient *K*_m_ (m^-1^) over the height of the vegetation (Hodges and Dallimore, 2015). In ELCOM, the total light extinction coefficient through the water column (*K*) is due to macrophyte presence (*K*_m_) and background water turbidity (*K*_w_). For model cells not occupied by macrophytes, *K* = *K*_w_, and the value of *K*_w_ was set to 1.5 m^-1^ based on PAR measurements at the LDS station (see Supplementary Simulations for details on *K*_w_ estimation). For model cells occupied by macrophytes, *K* = *K*_w_ + *K*_m_, and the value of *K*_m_ was set to 4 m^-1^ (Caraco and Cole, 2002). Although the biomass distribution of *P. crispus* is likely to differ with depth, specifically with greater biomass occurring toward the water surface (Kunii, 1982), we assumed for simplicity that *P. crispus* biomass is homogeneously distributed throughout the water column and thus applied a constant *K*_m_ with depth. *K*_w_ was assumed constant everywhere in the lake since visual inspection indicated similar water transparency across the lake.

While previous models of submerged macrophytes in shallow lakes account for the diffusion and dissipation of the surface-generated TKE by macrophytes, and thus require macrophyte density as an input parameter (Herb and Stefan, 2005b; Coates and Folkard, 2009), ELCOM assumes that no TKE penetrates through the canopy and does not require macrophyte density data. This assumption seems appropriate in dense canopies such as those commonly established by *P. crispus* since densely packed vegetation significantly reduces the TKE transported down into the deeper water (Coates and Folkard, 2009). Model cells were classified according to whether or not they were colonized by macrophytes. A continuous macrophyte bed was therefore represented by setting every model cell within the bed to be occupied by macrophytes (patch density *D*_m_ = 100%; see Supplementary Figure 1A). While vegetation height is a dimensional scalar variable in ELCOM, hereafter this parameter is expressed as vegetation height relative to water depth (*H*_m_), obtained by dividing plant height by total water depth at the LDS station. Water level data was obtained from a water level scale located at the lake outlet.

Bathymetric data of Lake Monger was collected in October 2011. The bathymetry was discretized using a uniform 5 m × 5 m horizontal grid with a vertical resolution of 0.1 m (**Figure 1**). The model was forced with meteorological data obtained from the LDS station (wind speed and direction), station T1 (rainfall, relative humidity, short wave radiation, and net total radiation) and Perth Metro (air temperature) and assumed uniform over the free surface of the model domain.

The model was calibrated at short and tall plant heights (*H*_m_ of 0.07 and 1, respectively), and validated at intermediate plant heights (*H*_m_ of 0.2 and 0.4) (see Supplementary Simulations for model set up). These four simulations were run over a period of 7 days because the model did not simulate macrophyte growth. During calibration and validation, the simulated water temperature at the LDS station was compared with the measured temperature records from each thermistor. To quantify model performance, the root-mean square error (RMSE) and the coefficient of determination (*r*^2^) were computed based on measurements at four or five discrete depths, depending on the lake’s water level. Once the model accurately represented the measured thermal structure at the LDS station at different plant heights (see Supplementary Figure 2), additional model simulations were set up and run over 7 days starting on 26 January 2015 to identify the submerged macrophyte attributes that impacted the onset of temperature stratification and horizontal water exchange (**Table 1**). The critical canopy relative height required for thermal stratification to occur was identified by varying *H*_m_ from 0 to 1 in increments of 0.1. To explore the relationship between macrophyte biomass and thermal stratification, we varied the light extinction coefficient since submerged vegetation biomass controls the extinction of light by its leaves (Owens et al., 1967; Baird et al., 2016). We used values of 4 and 2 m^-1^, following previous estimations for invasive and native submerged macrophyte species, respectively (Caraco and Cole, 2002). The relative roles of light attenuation versus canopy drag, for the onset of temperature stratification, were assessed in two further simulations by setting *K*_m_ or *C*_D_ to zero. Finally, the effect of a spatially fragmented canopy consisting of 25% macrophytes and 75% bare substrate was also tested. This was achieved by setting every fourth model cell within the macrophyte bed to be occupied by macrophytes (*D*_m_ = 25%; see Supplementary Figure 1B). To track the pathways of water movement in the model, an evenly distributed conservative numerical tracer was continuously released at all depths within the plant bed on 28 January 2015 between 10:00 and 20:00 h. This date was chosen because the wind conditions matched those observed on 4 November 2015, when the first diurnal field experiment took place. The tracer was released at a concentration of unity each time step (30 s), resulting in a maximum possible concentration of 1200 after 10 h.

**Table 1:** Simulations performed with ELCOM.

Simulated effect	*H*_m_	*D*_m_ (%)	*C*_D_	*K*_m_ (m^-1^)	*K*_w_ (m^-1^)
Changing canopy height	0–1^∗^	100	1	4	1.5
Reduced macrophyte light attenuation	1	100	1	2	1.5
No macrophyte light attenuation	1	100	1	0	1.5
No canopy drag	1	100	0	4	1.5
Reduced macrophyte patch density	1	25	1	4	1.5

^∗^*In the simulations that investigated the effect of canopy height on thermal stratification, H_*m*_ was varied from 0 to 1 to identify the critical canopy relative height required for thermal stratification to develop*.

*H_*m*_, relative macrophyte height; *D*_*m*_, macrophyte density; *C*_*D*_, macrophyte drag; *K*_*m*_, light extinction due to macrophytes, and *K*_*w*_, light extinction due to water and water constituents*.

### Thermal Stratification

To identify changes in the duration and strength of the thermal stratification simulated in ELCOM, we calculated the thermocline depth, which is the depth of maximum temperature gradient, using the software package “LakeAnalyzer” developed by Read et al. (2011) in MATLAB. A minimum temperature difference of 1°C was used to allow for noise removal in the temperature data. The water column was considered stratified when a thermocline formed and a temperature difference between surface and bottom waters of more than 1°C developed. The use of a 1°C threshold value seems appropriate in Lake Monger where strong temperature gradients (∼10°C m^-1^) are commonly observed during the *P. crispus* growing season.

## Results

### Critical Macrophyte Height Threshold for Thermal Stratification

#### Field Results

**FIGURE 2:**
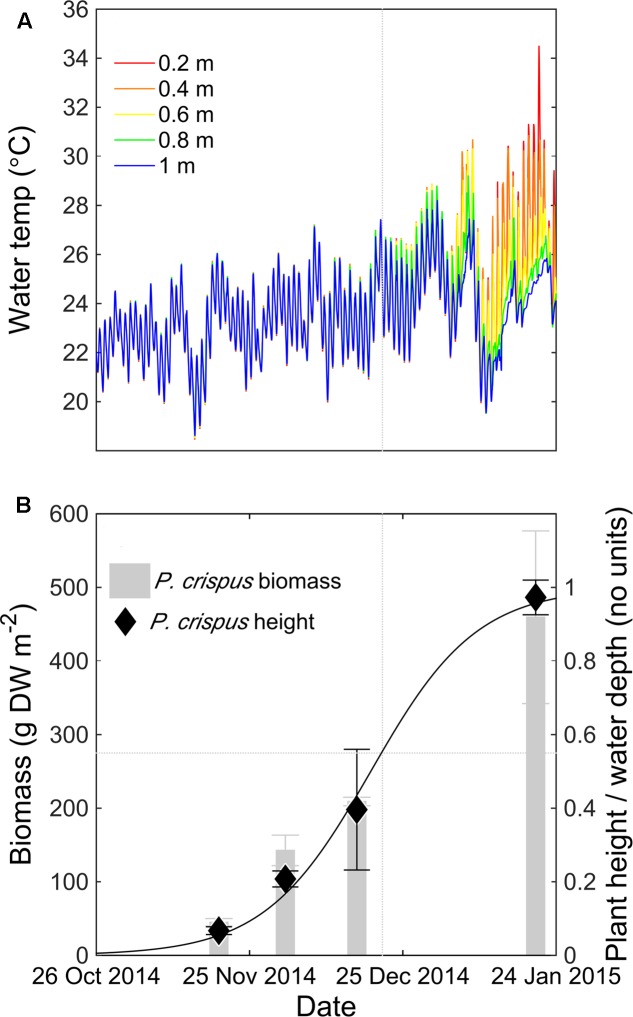
Time series of water temperature (°C) measured at the LDS station at 0.2 m (red), 0.4 m (orange), 0.6 m (yellow), 0.8 m (green), and 1 m (blue) depth for *Potamogeton crispus* growing season 2014–2015 **(A)**. *P. crispus* mean biomass (gray bars), standard deviation (gray error bars), relative height (diamonds), standard deviation of the height (black error bars) measured at the LDS station, and fitted logistic growth curve to changes in plant height over time for *P. crispus* growing season 2014–2015 **(B)**. The vertical dotted gray line indicates the time point where stratification forms (21 December 2014) and the horizontal dotted gray line indicates the biomass and canopy relative height corresponding to that time point.

In the first growing season, daytime thermal stratification was consistently observed at the center of the macrophyte bed after 21 December 2014, with maximum vertical temperature difference of 10°C between the water surface and sediment bed (0.8 m depth) (**Figure 2A**). The onset of temperature stratification coincided with an increase in both macrophyte height and biomass (**Figure 2B**). By fitting a logistic growth curve to the change in plant height over time (*r*^2^ = 0.98, *n* = 11), and comparing this curve to temperature data at the center of the macrophyte bed, we found that stratification began once the macrophytes occupied more than 50% of the water depth.

**FIGURE 3:**
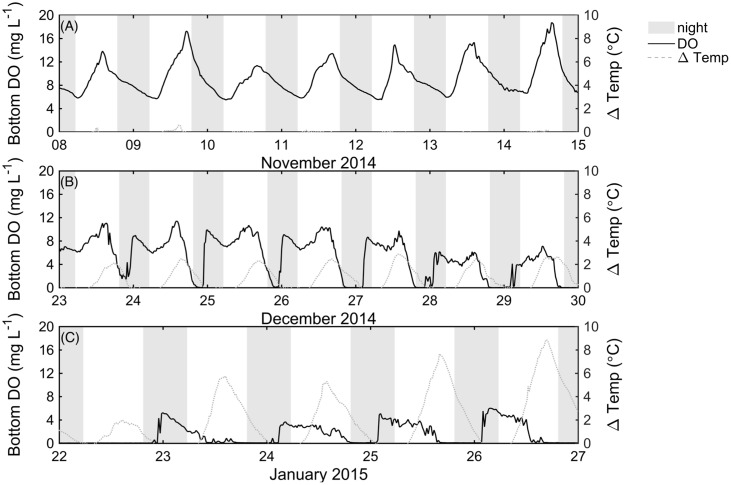
Measured bottom dissolved oxygen (mg L^-1^) in November 2014 **(A)**, and December 2014 **(B)** recorded with the Hydrolab DS 5X sensor at the LDS station, and measured bottom dissolved oxygen (mg L^-1^) **(C)** recorded with the Hydrolab Mini Sonde 4a at the LDS station in January 2015. In **(A–C)**, ΔTemp is the temperature difference between top and bottom thermistors, and shaded areas indicate nighttime.

Bottom DO levels at the center of the lake clearly declined as *P. crispus* biomass increased (**Figure 3**). In mid-November 2014, *P. crispus* canopies were short and no thermal stratification formed (**Figure 2B**). Bottom DO was ∼18 mg L^-1^ during the day, declining to ∼6 mg L^-1^ during night (**Figure 3A**). By December 2014, diurnal stratification with a maximum vertical temperature difference of approximately 3°C over 0.8 m had developed; at this time the daytime oxygen levels dropped to ∼11 mg L^-1^, and fully anoxic conditions were observed during the night (**Figure 3B**). In January 2015, strong thermal stratification developed (9°C over 0.6 m) and daytime DO levels remained below 6 mg L^-1^ reaching anoxic conditions before sunset (**Figure 3C**). Both in December 2014 and January 2015, anoxia lasted until water column mixing was established, suggesting that natural convection acts as a significant source of oxygen to the lakebed. Bottom redox potential at the LDS station in January 2015 followed a pattern similar to that of DO; it declined during night-time anoxia, reaching values of -450 mV and increased when surface and bottom waters mixed (Supplementary Figure 3).

#### Modeling Results

**FIGURE 4:**
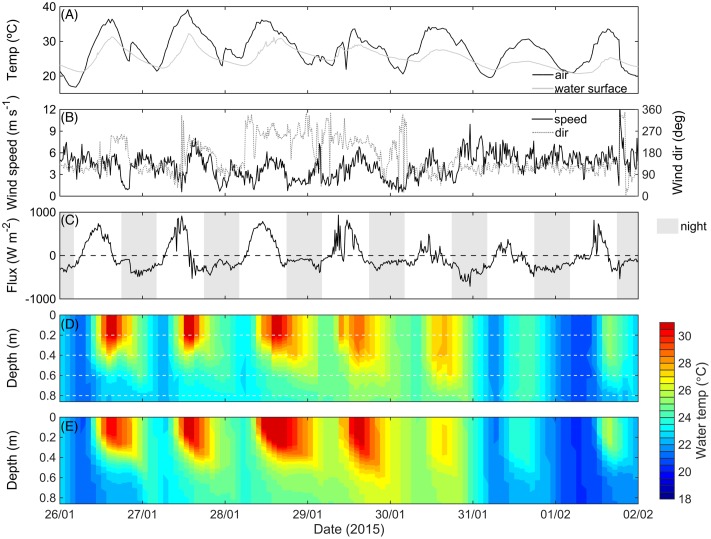
Measured air and water temperature at 0.2 m depth (°C) **(A)**, measured wind speed and direction (m s^-1^) **(B)**, net heat flux **(C)**, measured thermal structure **(D)**, and simulated temperature **(E)** at the LDS station at maximum relative plant height (*H*_m_ = 1) and biomass (459 g DW m^-2^ ± 25%) (see **Figure 2B**). Negative heat flux in **(C)** indicates cooling of the lake, and white dashed lines in **(D)** indicate the thermistor depths.

Given the importance of thermal stratification in controlling bottom oxygen dynamics at the center of the macrophyte bed, we used the ELCOM model to predict the conditions necessary for temperature stratification to occur. Air temperature, and wind speed and direction, are shown in **Figures 4A,B**, respectively. Net radiation flux from the air to the water, calculated from this data (Verburg and Antenucci, 2010), was typically positive during the day and negative during the night (**Figure 4C**). Measured thermal structure (**Figure 4D**) compared well with the ELCOM predictions of thermal stratification (**Figure 4E**; see also the Supplementary Figure 2D, RMSE = 0.93°C and *r*^2^ = 0.78). In both the measured and predicted thermal structure between 26 January 2015 and 2 February 2015, temperature stratification always occurred during the daytime. During the night, isothermal conditions also developed (**Figures 4D,E**) as the heat flux became negative (**Figure 4C**), suggesting that convective mixing was deepening the surface mixed layer.

**FIGURE 5:**
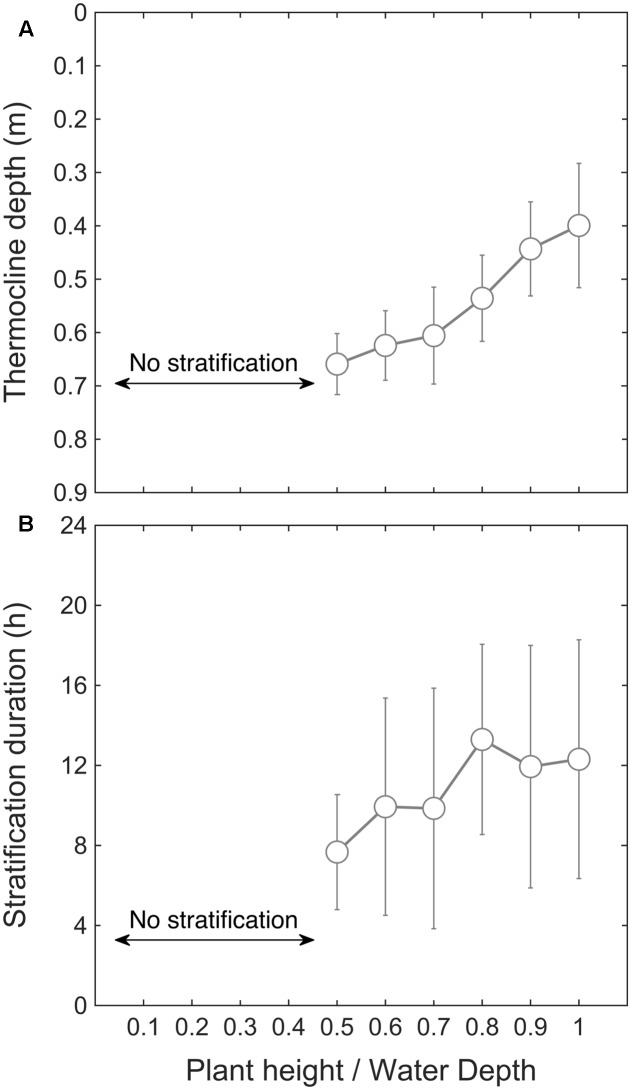
Simulated mean thermocline depth (m) versus canopy relative height **(A)**, and stratification duration (h) versus relative plant height **(B)**, obtained by varying *H*_m_ from 0 to 1 in ELCOM (see **Table 1**).

We then used the model to investigate how macrophyte height affects stratification. By varying the canopy relative height *H*_m_ from 0.1 to 1 (**Table 1**), the model predicted that a threshold canopy relative height of 0.5 is required for temperature stratification to develop (**Figure 5A**). Above this threshold height, the mean thermocline depth decreased as canopy height increased, from 0.66 ± 0.05 m (*H*_m_ = 0.5) to 0.40 ± 0.11 m (*H*_m_ = 1). In a similar way, the mean stratification duration increased with canopy relative height, from 7.7 ± 2.9 h per day (*H*_m_ = 0.5) to 12.3 ± 5.9 h per day (*H*_m_ = 1) (**Figure 5B**). In summary, the model results shown in **Figure 5** indicate that the submerged macrophytes induced thermal stratification once they occupied at least 50% of the water depth. This result is supported by field data collected in the *P. crispus* growing season in 2014–2015, in which temperature stratification developed after 21 December 2014, corresponding to a canopy relative height greater than 0.5 (**Figure 2**).

**FIGURE 6:**
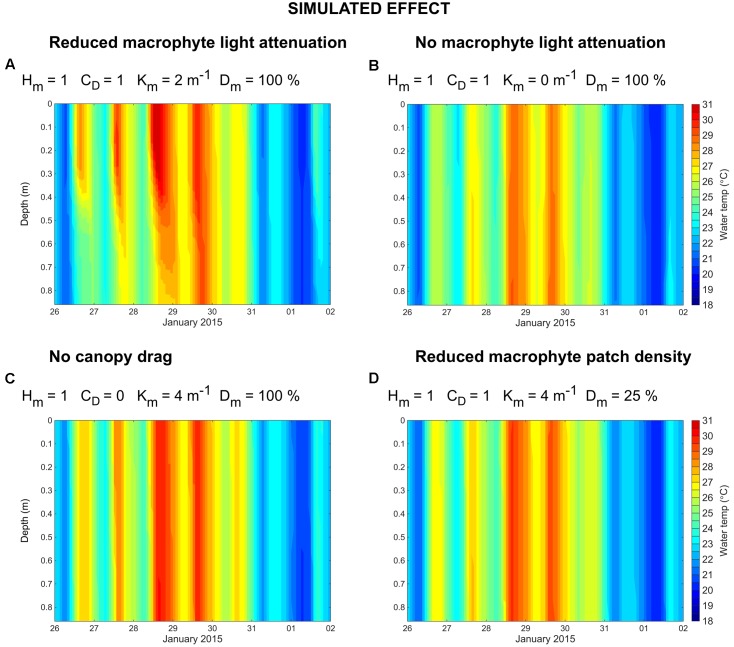
Simulated water temperature (°C) at the LDS station for the following simulated effects (see **Table 1**): reduced macrophyte light attenuation **(A)**, no macrophyte light attenuation **(B)**, no canopy drag **(C)**, and reduced macrophyte patch density **(D)**.

Additional model simulations were performed to investigate the relative importance of macrophyte attenuation of light, canopy drag and meadow continuity on the development of thermal gradients (**Table 1**). We found that, if light attenuation due to macrophyte presence is halved, thermal stratification may still develop (**Figure 6A**). However, if macrophytes provide no attenuation of light (**Figure 6B**), temperature stratification may not occur. Similarly, for a macrophyte bed that provides no canopy drag on local hydrodynamics (**Figure 6C**), or a macrophyte bed that is highly fragmented (25% macrophytes and 75% bare substrate; **Figure 6D**), thermal stratification does not occur. Together, these simulations suggest that (1) momentum loss induced by macrophyte canopy drag, (2) light absorption induced by macrophyte presence, and (3) a continuous (i.e., non-fragmented) macrophyte bed, may all be necessary for the development of macrophyte-induced water temperature gradients.

### Tall Canopies Reduce Horizontal Water Exchange

#### Field Results

**FIGURE 7:**
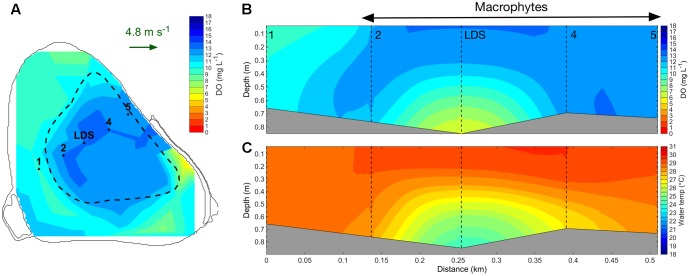
Interpolated surface maps of dissolved oxygen (mg L^-1^) produced with data collected by profiling the water column with the F-probe and averaging the first 0.25 m between 12:30 and 15:30 h on 28 January 2015 **(A)**. A southwest-northeast transect showing dissolved oxygen (mg L^-1^) **(B)** and water temperature (°C) **(C)** produced by interpolating profiles collected at stations 1, 2, LDS, 4, 5 between 13:11 and 13:34 h on 28 January 2015 are also shown. The location of the profiling points (1, 2, LDS, 4, 5) is indicated in **(A)**. The green arrow in **(A)** indicates the wind speed and direction. Dashed black line in **(A)** indicates the outer edge of the macrophyte bed. Dashed black lines in **(B,C)** indicate the location of each profiling station.

In addition to reducing vertical water exchange, tall canopies also substantially reduced horizontal water exchange (**Figure 7**). When the macrophytes reached the water surface on 28 January 2015, DO-rich surface water remained mostly within the macrophyte bed (**Figure 7B**), with lower concentrations occurring around the edges of the bed. Simultaneously, a substantial volume of DO-deplete water was confined to deep areas at the center of the macrophyte bed (**Figure 7B**). At the start of the 2015–2016 growing season, the macrophyte canopy had low height and therefore DO-rich surface water moved in the direction of the prevailing wind (Supplementary Figure 4). Later in this season, macrophytes reached the water surface, and wind-induced flows of DO-rich surface water were less evident (Supplementary Figure 5).

#### Modeling Results

**FIGURE 8:**
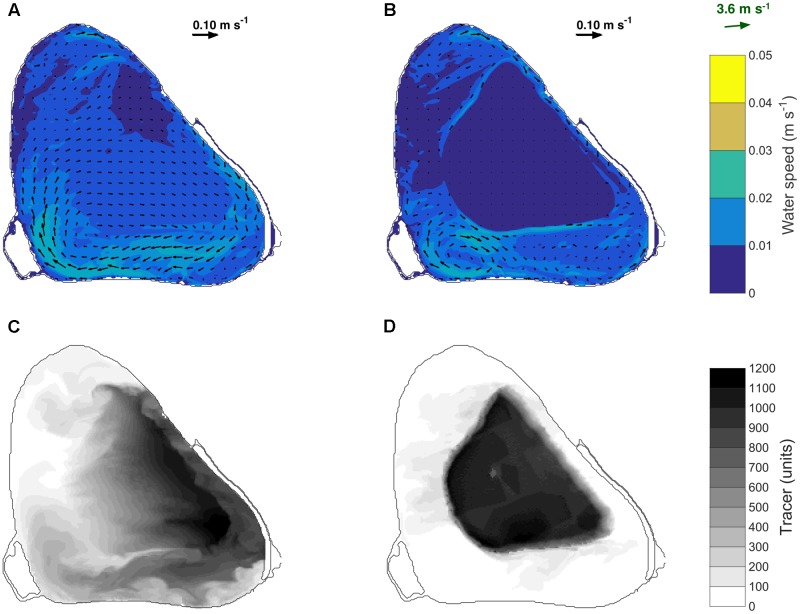
Depth-averaged velocity simulated on 28 January 2015 between 10:00 and 20:00 h for a simulated canopy relative height of 0.1 **(A)** and 1 **(B)**. Depth averaged distribution of the numerical tracer on 28 January 2015 at 20:00 h for a simulated relative canopy height of 0.1 **(C)** and 1 **(D)**. A tracer was continuously released in the model at a rate of 1 unit per 30 s between 10:00 and 20:00 h. The black arrows in **(A)** and **(B)** indicate the water speed and the green arrow indicates the wind speed and direction.

ELCOM simulations of a conservative tracer transport supported field observations as shown in **Figure 8**. For a canopy relative height of *H*_m_ = 0.1, the tracer was transported toward the east of the lake at a depth-averaged speed of 0.01 m s^-1^ (CV = 25%) (**Figures 8A,C**). However, if the macrophyte canopy reached the water surface, depth-averaged water velocities within the macrophyte bed were only 0.001 m s^-1^ (**Figure 8B**) and as a consequence the tracer remained mostly within the macrophyte bed, with water exchange only occurring along the edges of the plant bed (**Figure 8D**). Together with the observations described in Section “Field Results,” these model predictions indicate that, in addition to vertical mixing, as macrophytes approach the water surface, they also reduce horizontal water exchange between the center of the bed and the surrounding regions.

## Discussion

### Submerged Macrophyte Bed Impacts on Thermal Stratification and Oxygen

Our main finding is that dense aquatic canopies induce diurnal thermal stratification once they have occupied at least 50% of the water column. Specifically, we found that the onset of thermal stratification strongly controlled the diurnal patterns in DO close to the sediment-water interface. Shifting balances between oxygen production (photosynthesis), oxygen consumption (respiration) and transport processes induce changing oxygen levels over time. In the absence of thermal stratification, bottom-water DO followed typical diurnal cycles, increasing during the day due to photosynthesis and decreasing during the night due to respiration. However, when the plants occupied more than 50% of the water depth, anoxia manifested before sunset and lasted until night-time surface cooling induced vertical mixing of the water column, causing bottom-water DO to increase to almost daytime values. When the plants approached the water surface the water column remained stratified for longer, resulting in longer exposure to anoxia. Overall, as the plants grew in height, daytime bottom oxygen levels decreased, suggesting that the reduced light conditions due to increased self-shading influenced the balance between photosynthesis and respiration (Chimney et al., 2006). Convection-driven water transport from the lake edges to the macrophyte bed could also have contributed to increased bottom-water DO during nighttime, but this mechanism is unlikely to explain the increased oxygen levels in the macrophyte bed during the night (see Supplementary Text for details).

Our modeling results supported the field observation that thermal stratification developed once the macrophyte height is greater than 50% of the water depth. However, this threshold value depends on other plant attributes such as the canopy density and biomass. Halving the extinction coefficient to simulate a thinning of macrophyte biomass caused the mixed layer to deepen. This is in agreement with previous studies suggesting that light attenuation is critical for the development of thermal stratification (Dale and Gillespie, 1977). Thus, the critical canopy height for thermal stratification to develop is expected to vary with macrophyte density and water column transparency. Water depth may also influence the critical canopy height for thermal stratification to develop, and so this threshold height may differ in other ecosystems. In addition to the effect of light attenuation on vertical water exchange, macrophyte-induced thermal stratification requires a non-fragmented macrophyte bed (El Allaoui et al., 2016). Reducing the patch density caused the surface mixed layer to deepen to the lake bed, supporting previous observations that fragmented macrophyte beds allow for mixing to occur (Sand-Jensen and Mebus, 1996; El Allaoui et al., 2016). Moreover, the onset of thermal stratification required that the plants within the bed provide sufficient canopy drag forces (Herb and Stefan, 2004). In this study the relative importance of drag versus light attenuation for the onset of temperature stratification was analyzed in two further simulations. No thermal stratification developed in these simulations suggesting that both momentum loss and light absorption are necessary for the development of temperature gradients.

The modeling results also indicated that the strength of the stratification varies with macrophyte height. As the plants grew to the water surface, the thermocline depth decreased, supporting previous observations of stronger thermal gradients occurring in tall macrophyte beds compared with shorter beds (Herb and Stefan, 2005a). In the present study, stratification varied both vertically and across the plant bed. At maximum plant height (28 January 2015), stratification was less prominent at the bed edge compared with the center of the bed, so there may have been weaker oxygen gradients at the bed edge. Increased deposition of organic matter at the center of the macrophyte bed compared with the bed edges could also have contributed to cause low bottom-water DO in this region. However, at maximum plant height, higher levels of organic matter in the sediments were measured at the edge compared with the center of the macrophyte bed (45 and 33% OM, respectively, data not shown), supporting our suggestion that thermal stratification is the primary mechanism responsible for reducing the oxygen levels close to the bottom and center of the lake.

### Submerged Macrophytes Shape the Horizontal Variation in Water Quality

Our observation of weaker thermal gradients and higher oxygen levels at the edge compared with the center of the plant bed, at maximum plant height, suggested the presence of an exchange zone between the bed edge and the non-vegetated regions. This was supported by the interpolated surface maps of DO collected at tall canopy heights, in which DO levels were lower around the edge compared with the center of the bed. On the contrary, at low plant heights, surface DO and turbidity were transported in the direction of the wind, indicating that unlike tall beds, short beds are regularly flushed.

**FIGURE 9:**
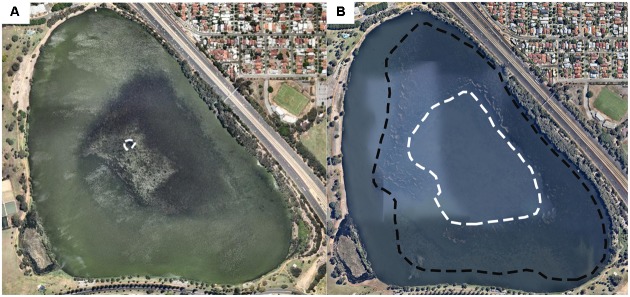
Aerial images of the lake obtained from http://maps.au.nearmap.com showing dieback at the LDS station (dashed white line). Date: February 8, 2015 **(A)** and March 4, 2016 **(B)**. The black dashed line in **(B)** shows the outer edge of the macrophyte bed. The extent of the ring in February 2015 was verified by field surveys.

Numerical modeling simulations also supported our field observations as follows. At low canopy heights (*H*_m_ = 0.1), the tracer was transported in the direction of the wind. However, as the plants reached the water surface, horizontal exchange with non-vegetated regions occurred in a narrow area around the bed edge (*H*_m_ = 1). Under these conditions, macrophyte-induced stratification is the key regulator of the bottom oxygen dynamics at the center of the plant bed. In addition to our observations at the center of the bed of reduced horizontal exchange, stronger thermal gradients and stronger exposure to anoxia, we observed central plant loss, with the edges of the bed persisting for longer (**Figure 9**). The extent to which this ring-shaped pattern is caused by the low oxygen recorded in this region remains unconfirmed.

### Thermal Stratification as a Negative Feedback Mechanism: A Driver of Plant Dieback?

Our results suggest that *P. crispus* can induce thermal stratification and reduce lateral transport of well-oxygenated waters into the center of the macrophyte bed, which in turn can create conditions that lead to *P. crispus* central dieback. Above a threshold height, submerged macrophytes induced thermal stratification of the water column, causing bottom-water anoxia at the center of the macrophyte bed. Anoxic conditions can cause plants to experience metabolic stress, which in turn can lead to declines in macrophyte biomass (Morris et al., 2004; Sand-Jensen et al., 2015). While some submerged macrophytes may survive bottom-water anoxia by supplying oxygen to below-ground tissue (Sand-Jensen et al., 1982; Lemoine et al., 2012), *P. crispus* cannot maintain oxygenated conditions within the sediments (Aldridge and Ganf, 2003; Boros et al., 2011) and thus may be more susceptible to anoxia exposure. Although the effect of water column anoxia on *P. crispus* survival has not yet been investigated, *P. crispus* can tolerate hypoxic conditions but is unable to survive under a combination of hypoxia and 0.5 mM of sulfide (Parveen et al., 2017). Under anoxic conditions, sulfide is likely to accumulate (Borum et al., 2005). In our study, indirect evidence of sulfide production was observed at maximum macrophyte biomass (black and slimy roots, a sulfidic odor and dark sediments). At critically low redox potentials (< -200 mV), which in our study developed at the sediment-water interface during thermal stratification, sulfide is expected to be produced (Stumm and Morgan, 1996; Andersen et al., 2017a).

Bottom-water anoxia could also have caused accumulation of other phytotoxins such as reduced nitrogen compounds (van Wijck et al., 1992), but it is unclear if this is responsible for the observed *P. crispus* dieback. In a separate study of Lake Monger, we observed that ammonium doubled during night-time stratification and reached concentrations of ∼0.030 mg L^-1^ (Vilas et al., 2017b). Since *P. crispus* shows antioxidative stress responses at ammonium concentrations ≥4 mg L^-1^ (Yin et al., 2016), it is highly unlikely that ammonium-induced toxicity would have caused the macrophytes to decline at the center of the bed. In addition, the plants could also have been exposed to the highly toxic gas ammonia. Conversion of ammonium to ammonia can occur under alkaline conditions (Nimptsch and Pflugmacher, 2007) such as those commonly established by *P. crispus*. Thus further investigations should be undertaken to assess the effect of these and other phytotoxins on *P. crispus* survival.

Anoxic conditions could also have inhibited periphyton grazers resulting in higher periphyton cover at the center of the macrophyte bed, which may have impaired macrophyte growth in this region (Scheffer, 2004). However, we did not measure periphyton cover and thus cannot identify if this mechanism was responsible for the plant dieback.

Regardless of the exact mechanism, we hypothesize that by inducing thermal stratification and reducing lateral transport of well-oxygenated waters into the center of the macrophyte bed dense canopies can cause conditions that can lead to their own decline in this region. This was the most plausible explanation for our observation of the ring-shaped pattern in spatial macrophyte distribution. Central dieback in aquatic plants has been observed elsewhere and attributed to demographic imbalance (Ruiz-Reynés et al., 2017) or gradual accumulation of sediment-derived sulfide in plant shoots (Borum et al., 2014), thus further investigations should be undertaken to identify the specific mechanism through which central anoxia can lead to ring-shaped pattern formation.

Alternative explanations for our observation of central dieback include: (a) the plants at the edges were younger and thus persisted for longer, (b) the plants at the center experienced post-flowering senescence (Chambers, 1982), (c) the plants at the center were light-limited, and (d) the plants at the center experienced thermal stress. Since the macrophytes initiated growth at the center and expanded outward, the plants at the edges were likely younger than those at the center. Thus, our observation of central dieback could be explained solely by the age of the plants. However, the area of the central dieback was smaller than the initial area colonized by the macrophytes (Supplementary Figure 7), which supports our hypothesis that thermal stratification and thus bottom anoxia may accelerate plant decline in this region. In addition, the plants were observed to produce flowers and turions across the plant bed, suggesting that weakening due to the production of reproductive structures may not explain the central dieback. The loss of macrophytes in the center of the macrophyte bed could not be attributed to light or temperature, which are thought to be involved in regulating the phenology of this species (Waisel, 1971; Valley and Heiskary, 2012). In both growing seasons, *P. crispus* plants grew to the water surface and thus were not light-limited. Moreover, minimal differences in turbidity levels were observed between the edges and the center of macrophyte bed (Supplementary Figure 5), suggesting that light reduction due to increased turbidity is unlikely to be responsible for the central dieback. Surface water temperatures also could not have triggered macrophyte senescence beginning in the center of the macrophyte bed, as follows. On 28 January 2015, a few days prior to *P. crispus* central dieback around the LDS station (**Figure 9A**), surface water temperatures were only 0.4°C higher at the center of the macrophyte bed compared to the eastern edge of the bed. On 7 January 2016, slightly higher water temperatures were measured at the eastern section of the lake (Supplementary Figure 5). However, macrophytes in this area persisted until March 2016 (**Figure 9B**), indicating that mechanisms other than temperature accelerated dieback at the center of the macrophyte bed. Light and temperature may still be ultimately responsible for lake-wide dieback of *P. crispus* by summer at our study site; however, these drivers cannot be responsible for the observed ring formation.

**FIGURE 10:**
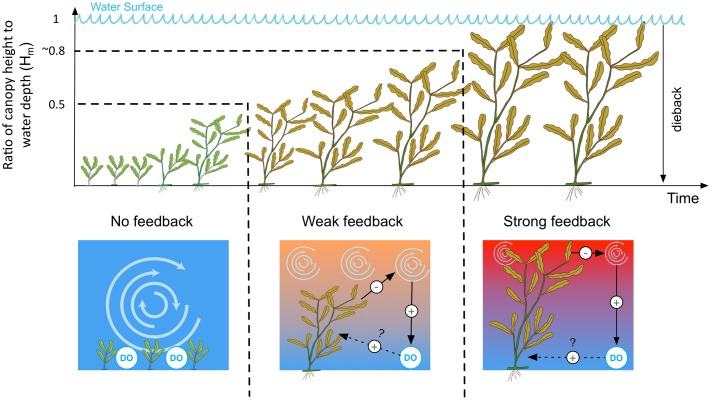
Conceptual model of the operation of the feedback on temperature stratification. DO, dissolved oxygen. The vertical temperature gradient is represented by red (hot) and blue (cold) colors. Positive and negative causal links are indicated with plus (+) and minus (–) signs, respectively. The dotted line indicates a proposed, though unconfirmed, causal link between dissolved oxygen and dieback suggested by our study (see Section “Thermal Stratification as a Negative Feedback Mechanism: A Driver of Plant Dieback?”).

In line with these observations we therefore propose a conceptual model for the negative feedback between macrophyte-induced thermal stratification and plant dieback (**Figure 10**). For clarification, we refer to the proposed feedback as negative because it is self-dampening and destabilizing (Maxwell et al., 2016) and to follow convention of previous research that explains feedback-induced ring-shaped patterns in ecology as a result of plant presence inducing environmental conditions that lead to plant dieback (Cartenì et al., 2012). In our case, the proposed negative feedback interaction between macrophyte density, stratification and plant loss is hypothesized to proceed as follows. Below a critical relative height threshold *H*_m_ of 0.5, submerged macrophytes do not stratify the water column, thus oxic conditions persist at the sediment-water interface. At intermediate relative canopy heights (*H*_m_ between 0.5 and ∼0.8), the thermal stratification occurs for less than half the day, reducing oxic conditions at the sediment-water interface. When the canopies approach the lake surface (*H*_m_ between ∼0.8 and 1), temperature stratification persists for more than half the day; under such conditions, lengthy anoxia is likely to develop close the lake bed. We hypothesize that these low bottom-water DO conditions then cause the macrophytes to accumulate stress that eventually leads to plant dieback.

Diurnal thermal stratification has long been observed in vegetated shallow lakes (Dale and Gillespie, 1977); however, the potential role of this mechanism in driving macrophyte distribution in shallow lake vegetation has been largely overlooked. Further research into the interaction between macrophyte distributions and thermal stratification in shallow lakes is especially pertinent because climate change is expected to increase the prevalence of thermal stratification in aquatic ecosystems (Wagner and Adrian, 2009).

## Conclusion

Thermal stratification, induced by submerged macrophytes, can reduce near-sediment oxygen concentrations at the center of the macrophyte bed, and may also potentially restrict the spatial patterns of these macrophytes. Fast-spreading macrophytes, such as the invasive *P. crispus*, may therefore engineer environmental conditions that paradoxically reduce their ability to invade. We suggest that interactions between macrophytes and thermal stratification may be ecologically significant in a variety of aquatic ecosystems.

## Author Contributions

MV performed the field experiments, analyzed the data, performed the modeling and wrote the manuscript. CM designed the field experiments and provided support with the 3D numerical modeling. MA and MH provided support with the modeling. CO proposed the diurnal field experiments and modeling scenarios. All authors interpreted the data and reviewed the manuscript. All authors approved the content of this manuscript.

## Conflict of Interest Statement

The authors declare that the research was conducted in the absence of any commercial or financial relationships that could be construed as a potential conflict of interest.

## Footnotes

1 http://www.bom.gov.au

2 http://maps.au.nearmap.com
