# Supplementary material for: Invasive Macrophytes Control the Spatial and Temporal Patterns of Temperature and Dissolved Oxygen in a Shallow Lake: A Proposed Feedback Mechanism of Macrophyte Loss
Source: Front Plant Sci. 2017 Dec 8;8:2097. doi: 10.3389/fpls.2017.02097 (PMC5727088; doi:10.3389/fpls.2017.02097)
Supplement: Supplementary file 1 [file Presentation_1.pdf]

Supplementary Material

Invasive macrophytes control the spatial and temporal patterns of temperature and dissolved oxygen in a shallow lake: A proposed feedback mechanism of macrophyte loss

Maria P Vilas*, Clelia L Marti, Matthew P Adams, Carolyn E Oldham, Matthew R Hipsey

*** Correspondence:** maria.vilas@research.uwa.edu.au

1 Supplementary simulations

Supplementary Table 1.

Simulation	1	2	3	4
Start date	14 Nov 2014	28 Nov 2014	12 Dec 2014	25 Jan 2015
End date	21 Nov 2014	4 Dec 2014	18 Dec 2014	2 Feb 2015
Canopy relative height	0.07	0.2	0.4	1

For each simulation the region of the lake that was colonized by macrophytes during the time of the simulations was obtained from the Nearmap® images. Data collected at the LDS was used to initialize water temperature distribution in the model within the macrophyte bed. The initial water level was obtained from a water level scale positioned at the lake outlet. The sediment reflectivity coefficient was varied from 0 to 1 (no units) and the extinction of the reflected light was varied from 0% to 50%. The sediment reflectivity coefficient and the extinction of the reflected light were set to 0.5 and 50%, respectively, as these values better represented the water temperature close to the bottom of the lake. The effect of macrophyte drag was tested varying C_D from 0.1 to 1, following values estimated by Abdelrhman (2003). The best model performance was achieved at $C_D = 1$, a value which has been shown to accurately represent the vegetation drag in a model seagrass meadow (Luhar and others 2010). Light attenuation due to macrophytes (K_m) was set to 4 m^{-1} (Caraco and Cole 2002) and the light extinction coefficient of the water was set to 1.5 m^{-1} (K_w) based on PAR measurements at the LDS station (collected on 7 January 2016, see Vilas and others 2017). The extinction coefficient was calculated as $I_z = I_0 e^{-Kz}$ (Kirk 1985), where I_z is the underwater light intensity at 0.9 m depth (z = 0.9), I_0 is the light intensity at the water surface, and $K = K_m + K_w$. Of note, we did not have light measurements during the simulated period to confirm whether the selected value of 1.5 m^{-1} is representative of field conditions both within and outside the macrophyte bed. However, we point out that our main interest was to develop a better understanding of the effect of submerged macrophytes on vertical and horizontal water exchange. Macrophyte density D_m was set to 100% cover given that no bare patches greater than 5 m x 5 m were observed in the field. The simulations time step was of 30 s.

2 Supplementary Figures

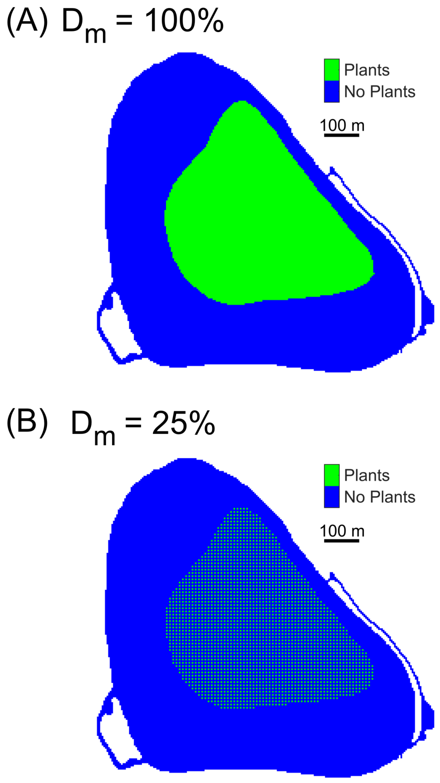

Supplementary Figure 1. Location of the submerged macrophytes simulated in ELCOM at two different patch densities (D_m): 100% **(A)** and 25% **(B)**.

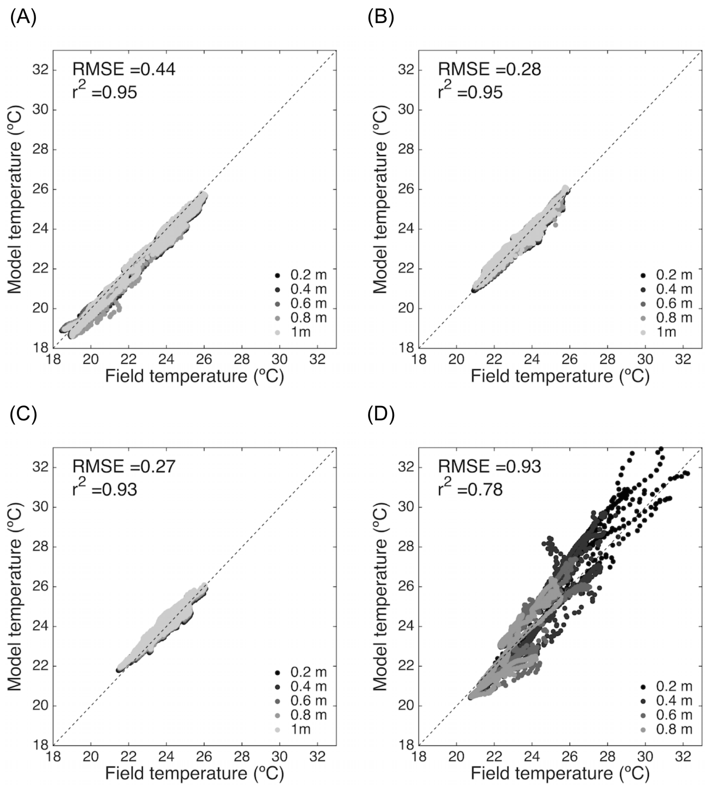

Supplementary Figure 2. Model water temperature (°C) versus field water temperature (°C) at the LDS station for simulation 1 **(A)**, 2 **(B)**, 3 **(C)** and 4 **(D)**.

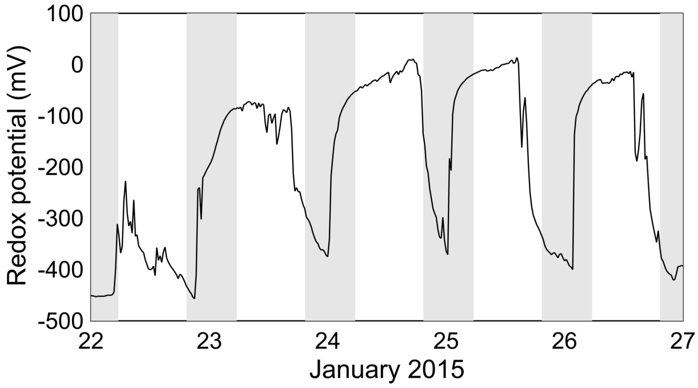

Supplementary Figure 3. Bottom redox potential (mV) recorded with the Hydrolab Mini Sonde 4a at the LDS station in January 2015.

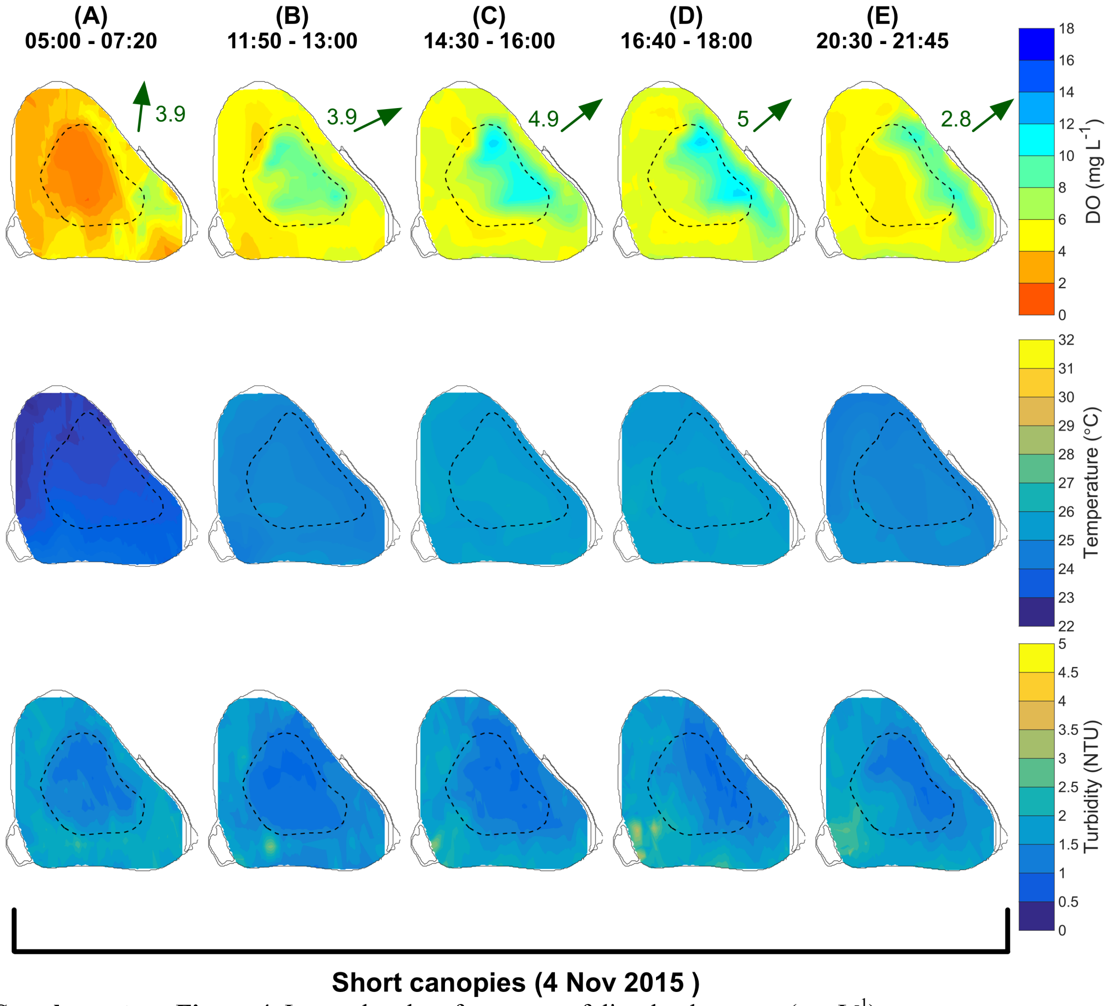

Supplementary Figure 4. Interpolated surface maps of dissolved oxygen (mg L^{-1}), water temperature (°C) and turbidity (NTU) produced with data collected by towing the F-probe at ~0.25 m depth on 4 November 2015 at different times of the day **(A, B, C, D and E)**. Mean wind speed (m s^{-1}) and direction for each map are also shown (green arrows). Dashed black line indicates the outer edge of the macrophyte bed.

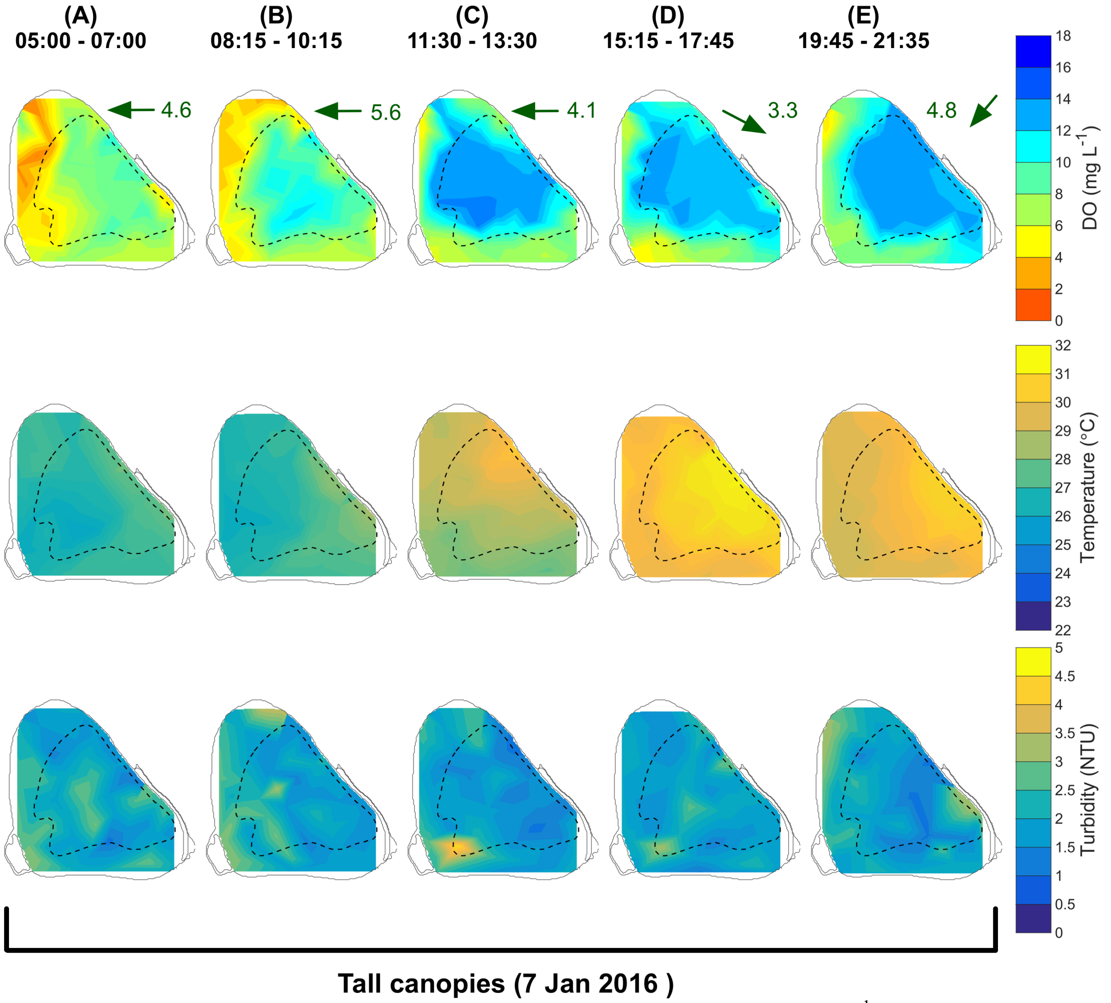

Supplementary Figure 5. Interpolated surface maps of dissolved oxygen (mg L^{-1}), water temperature (°C) and turbidity (NTU) produced with data collected by profiling the water column with the F-probe and averaging the first 0.25 m on 7 January 2016 at different times of the day **(A, B, C, D and E)**. Mean wind speed (m s^{-1}) and direction for each map are also shown (green arrows). Dashed black line indicates the outer edge of the macrophyte bed.

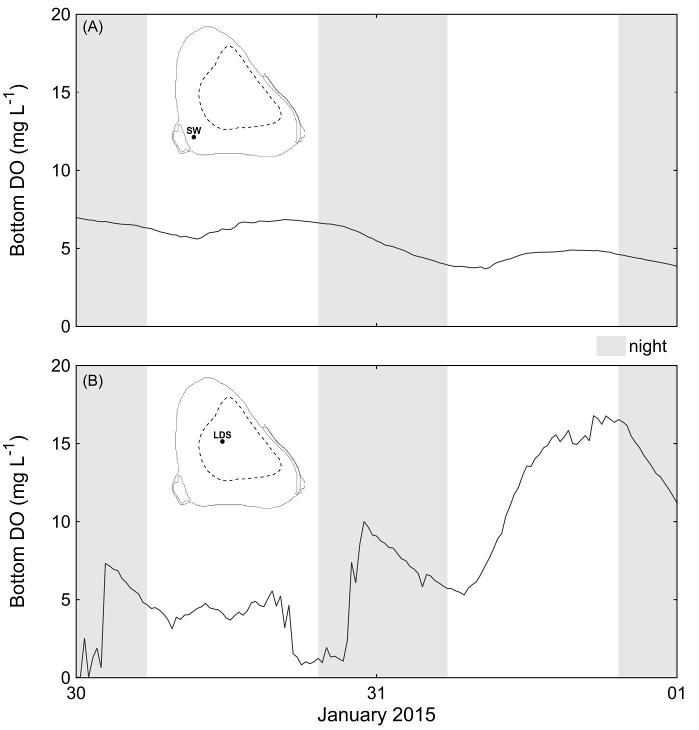

Supplementary Figure 6. Bottom dissolved oxygen (mg L^{-1}) between 30 January 2015 and 1 February 2015 at the SW station (A) and LDS station (B). Shaded areas indicate nighttime.

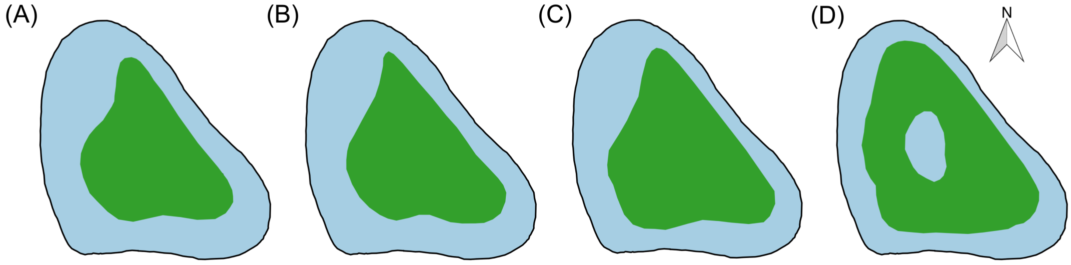

Supplementary Figure 7. The extent of the macrophyte bed produced from Nearmap aerial images (http://maps.au.nearmap.com) collected on 28 October 2013 **(A)**, 10 November 2013 **(B)**, 9 December 2013 **(C)** and 6 January 2014 **(D)**.

3 Supplementary text

In the manuscript, we stated that convection-driven water transport cannot explain, by itself, the increased oxygen levels in the macrophyte bed during the night. Here, we provide the justification for this statement.

Convection-driven water transport from the lake edges to the macrophyte bed could also have contributed to increased bottom-water DO during nighttime. During the day, submerged macrophytes shade the water column from incoming radiation, establishing a horizontal temperature gradient at the sediment bed between vegetated and non-vegetated areas (Ferris and Coates 1994). This horizontal gradient generates a near surface current from the lake edges to the macrophyte bed. During the night, vegetation reduces heat losses causing a reverse circulation between vegetated and non-vegetated areas as follows. As non-vegetated areas become colder faster than the vegetated areas, the colder water is then drawn along the bottom from non-vegetated areas to vegetated areas. Thus the observed increase in DO during the night could be a result of convective transport between vegetated and non-vegetated areas.

However, we point out that oxygen levels outside the macrophyte bed were lower than those recorded during nighttime mixing of the water column (see Supplementary Figure 6), thus this mechanism is unlikely to explain the increase in oxygen levels during the night.

4 References

Abdelrhman MA. 2003. Effect of eelgrass *Zostera marina* canopies on flow and transport. Mar Ecol Prog Ser 248:67–83.

Caraco NF, Cole JJ. 2002. Contrasting impacts of a native and alien macrophyte on dissolved oxygen in a large river. Ecol Appl 12:1496–509. http://onlinelibrary.wiley.com/doi/10.1890/1051-0761(2002)012[1496:CIOANA]2.0.CO;2/full

Ferris J, Coates MJ. 1994. The radiatively driven natural convection beneath a floating plant layer. Limnol Oceanogr 39:1186–94. http://doi.wiley.com/10.4319/lo.1994.39.5.1186

Kirk JTO. 1985. Effects of suspensoids (turbidity) on penetration of solar radiation in aquatic ecosystems. Hydrobiologia 125:195–208. http://link.springer.com/10.1007/BF00045935

Luhar M, Coutu S, Infantes E, Fox S, Nepf H. 2010. Wave-induced velocities inside a model seagrass bed. J Geophys Res Ocean 115:C12005.

Vilas MP, Marti CL, Oldham CE, Hipsey MR. 2017. Macrophyte-induced thermal stratification in a shallow urban lake promotes conditions suitable for nitrogen-fixing cyanobacteria. Hydrobiologia 806:411. http://link.springer.com/10.1007/s10750-017-3376-z
